# Interplay of self-organization of microtubule asters and crosslinking protein condensates

**DOI:** 10.1093/pnasnexus/pgad231

**Published:** 2023-07-13

**Authors:** Sumon Sahu, Prashali Chauhan, Ellie Lumen, Kelsey Moody, Karthik Peddireddy, Nandini Mani, Radhika Subramanian, Rae Robertson-Anderson, Aaron J Wolfe, Jennifer L Ross

**Affiliations:** Physics Department, Syracuse University, Syracuse, NY 13244, USA; Department of Physics, New York University, New York, NY 10003, USA; Physics Department, Syracuse University, Syracuse, NY 13244, USA; The Bioinspired Institute, Syracuse University, Syracuse, NY 13244, USA; Ichor Life Sciences, Inc., 2561 US Route 11, LaFayette, NY 13084, USA; The Bioinspired Institute, Syracuse University, Syracuse, NY 13244, USA; Ichor Life Sciences, Inc., 2561 US Route 11, LaFayette, NY 13084, USA; Lewis School of Health Sciences, Clarkson University, 8 Clarkson Avenue, Potsdam, NY 13699, USA; Physics Department, University of San Diego, San Diego, CA 92110, USA; Massachusetts General Hospital, Boston, MA 02115, USA; Massachusetts General Hospital, Boston, MA 02115, USA; Physics Department, University of San Diego, San Diego, CA 92110, USA; The Bioinspired Institute, Syracuse University, Syracuse, NY 13244, USA; Ichor Life Sciences, Inc., 2561 US Route 11, LaFayette, NY 13084, USA; Lewis School of Health Sciences, Clarkson University, 8 Clarkson Avenue, Potsdam, NY 13699, USA; Physics Department, Syracuse University, Syracuse, NY 13244, USA; The Bioinspired Institute, Syracuse University, Syracuse, NY 13244, USA

**Keywords:** liquid–liquid phase separation, microtubule asters, aging, self-organization, MAP65-1

## Abstract

The cytoskeleton is a major focus of physical studies to understand organization inside cells given its primary role in cell motility, cell division, and cell mechanics. Recently, protein condensation has been shown to be another major intracellular organizational strategy. Here, we report that the microtubule crosslinking proteins, MAP65-1 and PRC1, can form phase separated condensates at physiological salt and temperature without additional crowding agents in vitro. The size of the droplets depends on the concentration of protein. MAP65 condensates are liquid at first and can gelate over time. We show that these condensates can nucleate and grow microtubule bundles that form asters, regardless of the viscoelasticity of the condensate. The droplet size directly controls the number of projections in the microtubule asters, demonstrating that the MAP65 concentration can control the organization of microtubules. When gel-like droplets nucleate and grow asters from a shell of tubulin at the surface, the microtubules are able to re-fluidize the MAP65 condensate, returning the MAP65 molecules to solution. This work implies that there is an interplay between condensate formation from microtubule-associated proteins, microtubule organization, and condensate dissolution that could be important for the dynamics of intracellular organization.

**Significance Statement**

The fundamental organizational principles used by the cell to organize its interior space are still not fully elucidated. Two important mechanisms for cellular self-organization include liquid–liquid phase separation of protein species to form membraneless organelles and self-organization of cytoskeletal filaments into larger-scale arrangements such as the actin cortex and microtubule mitotic spindle. Here, we demonstrate that microtubule-crosslinking proteins, MAP65 and PRC1, can self-assemble into liquid-like condensates that can catalyze spatially controlled nucleation and growth of microtubule asters with control over the aster organization. Microtubules grown from gel-like condensates can reverse the gelation, controlling the material properties. This interplay between protein condensates and the cytoskeleton could allow for dynamic reorganization of the cell interior in space and time.

Intracellular organization from the molecular to cellular scale is still an unsolved mystery requiring biological, chemical, and physical approaches. Within the last decade, the cell biological field has found a new appreciation for the ability of proteins to reversibly form condensed phases termed liquid–liquid phase separation (LLPS) (1). These condensates can act as membraneless organelles, transiently bringing together molecular species to perform essential cellular activities (2). The reversibility allows these activity centers to form when needed and disperse, and these changes can be linked to Circadian rhythms (3). Such condensates can have liquid-like and gel-like material properties, perform their functions, and still reversibly dissolve (4).

Prior cellular organization studies have focused predominantly on the cytoskeleton, which is a filamentous scaffolding system required for cell morphology, cell division, and cell motility (5). Many studies on microtubule self-organization have discovered that these stiffer cytoskeletal filaments act as nematic species that prefer to align side-to-side (6–9). Such activity has been shown to be important for the spindle shaped organization of microtubules in the cell division machinery (10, 11). In cells, microtubules are often specifically nucleated and grown from microtubule-organizing centers (MTOCs) that spatially and temporally control the organization of microtubules. Many MTOCs are synonymous with centrosomes that contain the centriole, a complex made of gamma tubulin and other proteins organized into a barrel shape that can template microtubules directly. Yet, not all cells have centrosomes, and centrosomes have been shown to be dispensable for even the most important processes, including mitosis (12, 13). Further, microtubule nucleation can also occur from other organelles, such as the Golgi complex (14) and from the sides of microtubules in the mitotic spindle (15).

Recently, it has been shown that condensed, liquid droplets of microtubule-associated proteins (MAPs), such as tau, TPX2, CAMPSAP2, and BuGZ, Abl2, and +Tip proteins, can form condensates via LLPS and nucleate microtubules when free tubulin is added to these condensates (16–23). Thus, the relatively new cellular organization principle of LLPS may have bearing on traditional organization schemes that use the cytoskeleton.

MAP65-1 (MAP65) is a microtubule-associated protein crosslinker from the PRC1/MAP65/Ase1 family that can promote nucleation of the microtubules (24–26). Here, we demonstrate a possible mechanism for microtubule spatial organization by MAP65-induced co-localization and nucleation via LLPS. We show that both MAP65 and PRC1 can condense into liquid-like droplets in vitro without added crowding agents. We quantify the material properties of the MAP65 condensates and determine that the mobility of MAP65 slows over time causing a transition to a more gel-like phase. When free tubulin is added, both the liquid- and gel-like condensates are capable of concentrating tubulin dimers. The concentration is high enough to nucleate and grow microtubules into spindle-like tactoids or asters. The number of microtubule projections in the aster is directly proportional to the diameter of the droplets, which is directly controlled by the concentration of MAP65. Interestingly, we observe that the MAP65 in gelated condensates can reversibly dissolve due to the presence of the microtubule asters. Thus, while the MAP65 condensates can control the microtubule organization, the microtubules can in turn control the condensate properties. Collectively, this suggests a feedback system for controlling both condensates and microtubules in space and time that could be important for intracellular organization.

## Results

### MAP65 and PRC1 can condense via liquid–liquid phase separation

**Fig. 1.**
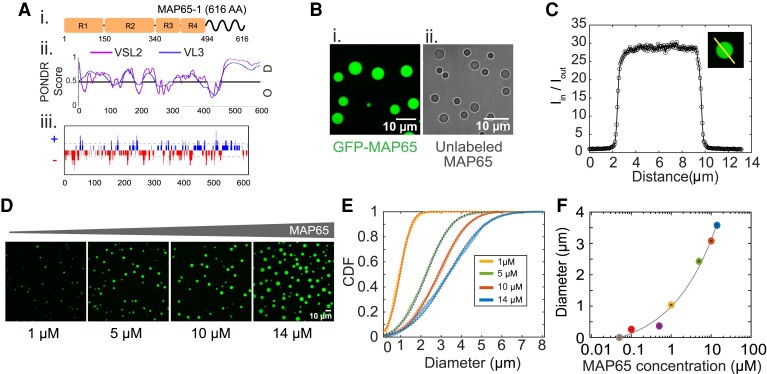
MAP65 can form liquid condensates. A) (i) Cartoon of MAP65 sequence regions. $R_{1},R_{2},R_{3},$ and $R_{4}$ boxes represent spectrin domains. Tail domain is an IDR. (ii) Predicted disorder probability from PONDR algorithms, VSL2 (magenta) and VL3 (blue). Score >0.5 is considered disordered. (iii) Predicted net charge per residue (NCPR) distribution from CIDER with positive charges (blue) and negative charges (red). B) Images of phase separated MAP65 in (i) confocal fluorescence or (ii) transmitted light microscopy. Scale bars are 10 μm. C) Quantification of the partition coefficient, p, from the intensity profile of a confocal slice through the center of a droplet. Intensity is normalized so that the background level is one. D) Confocal fluorescence images of droplets showing diameter increases with MAP65 concentration for 1, 5, 10, and 14 μM. Scale bar is 10 μm. E) Cumulative distribution functions of droplet diameter for 1 μM (yellow, left-most curve), 5 μM (green, second curve), 10 μM (red, third curve), and 14 μM (blue, right-most curve) MAP65 concentrations. Each is fit to a normal distribution to find the mean (same as median) and standard error of mean. F) Median diameter of droplets for 50 nM, 100 nM, 500 nM (purple), 1 μM, 5 μM, 10 μM, and 14 μM MAP65 concentrations. Data fit with an shifted power law function, $y=ax^{b}+c$. The best fit parameters are given in the Supplementary material.

We use full length WT MAP65-1 (MAP65) from *Arabidopsis*, a member of the Ase1/PRC1/MAP65 family of microtubule crosslinking proteins. Full-length MAP65 has four rod and spectrin domains along the sequence and intrinsically disordered regions (IDRs) in the C-terminal tail (Fig. 1Ai) as predicted by two different PONDR algorithms (VSL3 and VL3, Fig. 1Aii) (27). The sequence has locally concentrated positive and negative charge regions, predicted by CIDER net charge per residue (NCPR), in the IDRs (Fig. 1Aiii). These molecular properties are known markers for phase separation, making MAP65 a good candidate to form condensates.

We observed that purified, full-length MAP65 forms condensates via LLPS at $22\pm 1^{\circ }C$ in a typical tubulin polymerization buffer, PEM-80 (80 mM PIPES, 1 mM EGTA, 2 mM MgCl${}_{2}$) at pH 6.8 (Fig. 1B). For confocal imaging, 10% of MAP65 was tagged with green fluorescent protein MAP65 (GFP-MAP65), allowing us to visualize round droplets using confocal fluorescence imaging (Fig. 1Bi). To ensure that GFP is not driving condensation, we imaged unlabeled full-length MAP65 using transmitted light microscopy in the same conditions and found that unlabeled MAP65 also formed condensates (Fig. 1Bii).

From confocal imaging, MAP65 droplets appear as a single condensed phase that is composed of a MAP65-rich domain separated from the surrounding solution. There are no inhomogeneities or higher density regions within the droplet. We quantified the partition coefficient, *p*, to determine the relative difference in concentration between the droplet inside and outside using the intensity profile through a single confocal slice in the middle of the droplet, $p=I_{\mathrm{in}}/I_{\mathrm{out}}$ (Fig. 1C), where $I_{\mathrm{in}}$ and $I_{\mathrm{out}}$ are the average intensity inside and outside the condensate, respectively. We find that p=20.5±0.3 when the MAP65 concentration is 14 μM (mean±SEM, N=2466 droplets). We find that the partition coefficient of droplets increases with MAP65 concentration, saturating above 10 μM (Supplementary material, Fig. S2).

As the MAP65 concentration increases, the droplet diameter increases (Fig. 1D and E). We find that droplets can form when there is as little as 100 nM MAP65 (Fig. 1F, Supplementary material, Fig. S2). Fitting the diameter data as a function of MAP65 concentration using a shifted power law, we deduce that the critical concentration for LLPS of MAP65 is 48 nM (Fig. 1F, Supplementary Material, Fig. S2). This low concentration is likely within the physiological range for MAP65 in cells, implying that small droplets may be able to form in cells. As a comparison, PRC1 is found to be 125 nM in HeLa cells (28).

MAP65 is the plant analog of the PRC1 protein from mammalian cells that organizes microtubules during mitosis. Full-length PRC1 has similar domains as MAP65, including spectrin domains and an intrinsically disordered tail, similar charge distributions, and similar predictions for unstructured regions. Like MAP65, PRC1 can also form condensates (Supplementary material, Fig. S3).

Protein condensates are sensitive to environmental parameters such as temperature and ionic strength of the environment (1). The theoretical isoelectric point (pI) of MAP65 is 5.07 estimated using ExPasy Protparam, suggesting that MAP65 is overall negatively charged at pH 6.8, but the charge distribution has positive and negative charges distributed along the length with a positively charged tail (Supplementary material, Fig. S4). The addition of salt inhibits MAP65 and PRC1 condensate formation, reducing both the condensate number density and droplet size (Supplementary material, Fig. S4).

### Material properties of MAP65 condensates

**Fig. 2.**
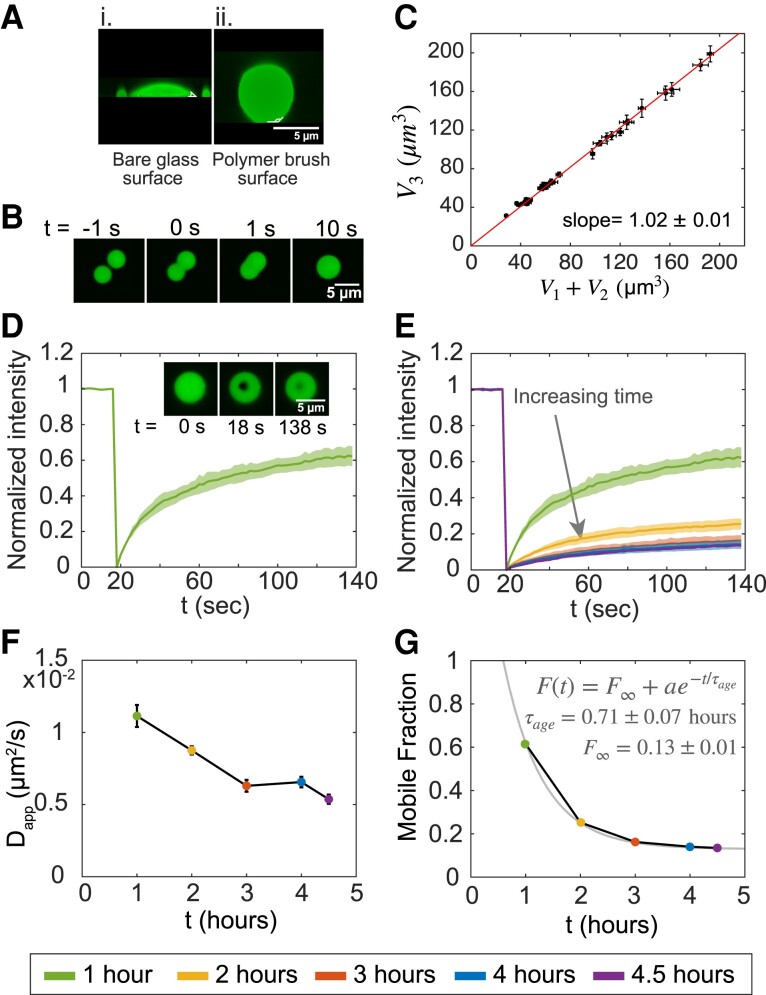
MAP65 droplet materials properties. A) Confocal images of droplets in the *X*–*Z* plane for MAP65 condensates (i) on bare glass, and (ii) on a polymer brush surface. Scale bar is 5 μm. B) Images of droplet fusion over time where t=0 s indicates the frame where fusion starts. Scale bar is 5 μm. C) Plot of the volume of the sum of the droplets’ volume before fusion, $V_{1}+V_{2}$, and after fusion, $V_{3}$ (n=31). The best fit slope is 1.02±0.01, indicating that volume is conserved. D) Quantification of FRAP normalized such that the initial intensity is one and the minimum intensity is zero with average (dark green line) and standard deviation (green shaded region) displayed (N=8). Image inset: FRAP sample data with droplet before bleach (t=0 s), at bleach (t=18 s), and after recovery (t=138 s). Scale bar is 5 μm. E) Normalized FRAP recovery curves for each five time points for maturation times of 1 h (N=8, green, upper-most curve), 2 h (N=10, yellow, second curve from top), 3 h (N=9, red, third curve), 4 h (N=10, blue, fourth curve), and 4.5 h (N=6, purple, bottom curve). The shaded regions indicates the standard deviation around the mean. F) Apparent diffusion coefficients calculated from FRAP recovery time, $\tau_{1/2}$, plotted against maturation time. The bars indicate a 95% confidence interval. G) Mobile fraction data show exponential decay plotted against maturation time. The exponential decay fit (grey line) yields a characteristic aging time $\tau_{\mathrm{age}}=0.71\pm 0.07$ h with mobile fraction at long time, $F_{\infty }$, to be ∼13%.

Condensed proteins can have liquid-like or gel-like properties that can change and reverse depending on the environment and other factors. We find that MAP65 condensates are able to wet the glass cover slip surface when the glass is untreated making a contact angle of $\theta ∼50^{\circ }$ (Fig. 2Ai). Wetting the surface inhibits further experiments such as fusion experiments to characterize condensate size and material properties. To reduce wetting, we precoat the cover glass surface with a polymer brush, Pluronic-F127, as previously described (9, 29). The polymer brush surface prevents protein adsorption and facilitates surface dewetting with an approximate contact angle $\theta ∼130^{\circ }$ (Fig. 2Aii). This surface coating allows MAP65 condensates to diffuse on the surface.

When two liquid-like MAP65 condensates come close and touch each other by thermal motion, they fuse into a single droplet rapidly (Fig. 2B). Imaging confocal images over time during merging events, we quantify the droplet volumes before and after fusion. Plotting the volume of the merged final droplet, $V_{3}$, as a function of the summed volume of the two droplets before merging, $V_{1}+V_{2}$, we find that the slope is one, as expected if the volume is constant before and after droplet merging (Fig. 2C).

We find that temperature affects MAP65 condensation (Supplementary material, Fig. S4). At low temperature $4^{\circ }C$, many small droplets form. At room temperature, the droplets are large and round, and at high temperature, $37^{\circ }C$, droplets are nonspherical and adhered to one another indicating nucleation and growth of condensates with incomplete merging events between multiple droplets that are no longer liquid. These data imply that higher temperatures cause a transition from liquid- to gel-like condensates. For PRC1 at $37^{\circ }C$, condensates are still round and liquid-like at higher temperatures (Supplementary material, Fig. S4). PRC1 is found in mammals that have a higher body temperature, implying that droplets viscosity may be evolutionary tuned between plant and mammalian versions of the protein.

We use fluorescence recovery after photobleaching (FRAP), we test the mobility of the molecules within a condensate using a 405 nm laser to photobleach a small region within the droplet and image recovery (Fig. 2D). We quantify the fluorescence of the region over time, as described in the supplement (Supplementary material, Fig. S1). The average fluorescence intensity of the spot rapidly recovers to 60%–65% within a 2 min timescale (Fig. 2D). These data indicate that the MAP65 in the condensate is mobile and liquid-like.

Several proteins such as FUS, hnRNPA1, hnRNPA2, EWS, TAF15, and FIB1 are reported to form condensates that become gel-like with time (30–39). The change in the material properties from fluid-like to viscoelastic gels has been referred to as gelation, aging, hardening, or maturation of droplets. Despite the negative connotation of a term like “aging,” gelation is often reversible, does not necessarily lead to aggregation, and may be a biologically relevant state for some condensates (2, 4).

Using the point photobleaching, we measure the molecular mobility inside droplets as the droplet sample matures over 4.5 h (Fig. 2E). We quantify and fit the data to find the recovery halftime, $\tau_{1/2}$ (Supplementary material, Eq. [1]). The $\tau_{1/2}$ values show an upward trend with the time that characterizes the reduced mobility of condensate maturation (Table 1). An hour after mixing, $\tau_{1/2}=27.16\pm 1.86$ s, which jumps up two-fold at 4.5 h, which is $\tau_{1/2}=56.33\pm 3.38$ s (Table 1).

**Table 1.** MAP65 condensate data over maturation time.

Time (h)	$\tau_{1/2}(s)$	*D*_app_ × 10^−2^μ	Mobile fraction	Approx. viscosity (Pa-s)
1	27.2±1.9	1.11±0.07	0.62	4.7
2	34.6±1.1	0.88±0.03	0.25	5.8
3	48.0±3.1	0.63±0.04	0.16	8.1
4	46.1±2.6	0.66±0.04	0.14	7.8
4.5	56.3±3.4	0.54±0.03	0.13	9.5

The recovery halftimes are used to estimate the apparent diffusion coefficients, $D_{\mathrm{app}}$: $D_{\mathrm{app}}∼\frac{r^{2}}{\tau_{1/2}}$, where *r* is radius of the photobleached region (40, 41); r∼0.55μm for our experiments (Supplementary material, Fig. S1). The $D_{\mathrm{app}}$ decreases as the droplets gelate over hours (Fig. 2F, Table 1). Using a second method (42), we confirm the $D_{\mathrm{app}}$ values, implying the method is robust (Supplementary material).

We quantify incorporation of MAP65 into droplets from the solution by photobleaching whole droplets at different maturation times (Supplementary material, Figs. S5 and S6). We quantify and fit the radially averaged intensity profile of each photobleached droplet using a 1D diffusion equation (Supplementary material, Figs. S5 and S6). The internal diffusion co-efficient, $D_{\mathrm{in}}$ is estimated from the best fit (Supplementary material, Fig. S6). We find the diffusion coefficient inside the droplet $D_{\mathrm{in}}$ is similar to the apparent diffusion coefficient $D_{\mathrm{app}}$ (Table 1, Supplementary material, Table S1, Fig. S6).

The diffusion of protein molecules is coupled to the viscosity of the environment by the Stokes–Einstein equation using the hydrodynamic radius of the polymer for the particle radius. The hydrodynamic radius of MAP65, was reported to be ∼42 Å (25). At early times, the estimated viscosity is η∼4.7 Pa-s or 4,670 mPa-s, which is similar to the viscosity of honey. For comparison, the dynamic viscosity of water at $22^{\circ }C$ is 0.95 mPa-s (Table 1).

Using the plateau value of the FRAP recovery curve, we estimate the mobile fraction of the molecules in the droplets. The mobile fraction dropped from ∼60% after 1 h of maturation, to ∼14% after 4.5 h. The reduction in mobile fraction was fit to an exponential form to reveal that a small fraction (∼13%) of molecules remains mobile as the droplets gelate (Fig. 2F).

Quantifying the rate of droplet merging reveals the surface tension of LLPS condensates (full method in Supplementary Material). When droplets are liquid-like, we plot the time of merging against the characteristic size of the merging droplets, and the slope is proportional to the ratio of viscosity to surface tension (Supplementary material, Fig. S7A). Using the viscosity found from $D_{\mathrm{app}}$, the surface tension of liquid-like droplets is γ∼12.3μN/m in the first hour, which is similar to other protein condensates (43). At long times, droplets appear unable to merge on the timescale of the experiments (Supplementary material, Fig. S7B), as expected for viscoelastic gels.

### MAP65 condensates control microtubule aster organization

Protein condensates are used to organize the cell interior by creating regions of high local concentration to control specific reactions. Condensates of microtubule-associated proteins can control cytoskeletal networks by increasing the local concentration inside the condensate above the critical concentration to organize the nucleation and growth of cytoskeletal filaments (16, 18, 19, 44). To test if MAP65 condensates can concentrate tubulin to cause the nucleation of microtubules, we combine free tubulin with MAP65 droplets.

**Fig. 3.**
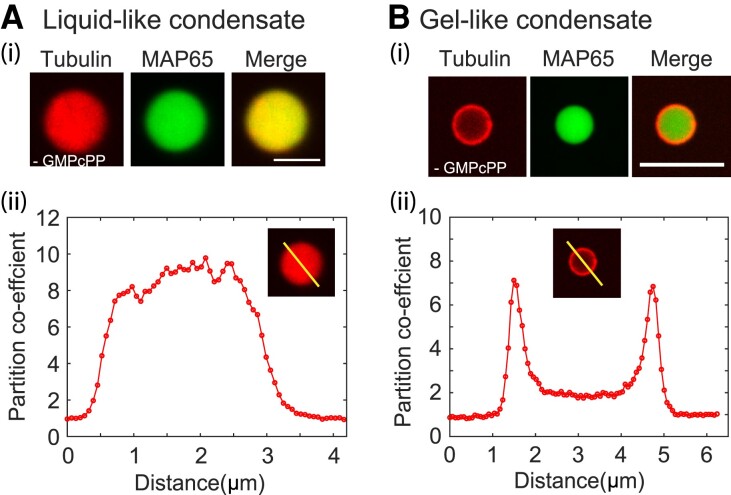
Tubulin colocalization and microtubule organization with MAP65 condensates. A) Tubulin co-localizes with MAP65 liquid droplets. (i) Representative confocal image of rhodamine tubulin (red, left) and GFP-MAP65 (green, center) and overlay (merge, right) in a liquid-like droplet. Scale bar is 5 μm. (ii) Intensity scan through the droplet in the rhodamine channel normalized so that the intensity outside is one. B) Tubulin co-localizes with MAP65 matured droplets. (i) Representative confocal images of rhodamine tubulin (red, left) and GFP-MAP65 (green, center) and overlay (merge, right) in a gel-like droplet. Scale bar is 5 μm. (ii) Intensity scan through the droplet in the rhoamdine channel normalized so that the intensity outside the droplet is one.

First, we examine the accumulation of tubulin into condensates by adding a low concentration of tubulin, [TUB]=3.75μM without nucleotide to preformed MAP65 condensates made with 10 μM MAP65. Droplets are examined 30 min after adding tubulin to allow the tubulin to equilibrate through diffusion (Fig. 3).

An intensity scan of a confocal slice through the center of the droplet is used to determine the partition coefficient (Fig. 3A). We find that the average partition coefficient for tubulin inside compared to outside the droplet is p=7.2±0.3 (mean±SEM, N=55 droplets). Given the total concentration of tubulin added, we estimate that the concentration inside these droplets is ∼26 μM high enough to nucleate microtubules locally, if we had provided guanosine nucleotide to the system.

When tubulin without nucleotide is added to MAP65 condensates that have gelated, tubulin has a higher concentration at the condensate surface and less concentrated in the droplet center (Fig. 3B). The tubulin concentration at the edge is p=4.8±0.2 (mean±SEM, N=26 droplets). The intensity of tubulin inside the center of the droplet is 2.1±0.1 (mean±SEM, N=26 droplets) times higher than the concentration outside of the droplet. We estimate that the tubulin concentration in the ring is ∼18 μM, which should be able to nucleate microtubules when nucleotide is present.

**Fig. 4.**
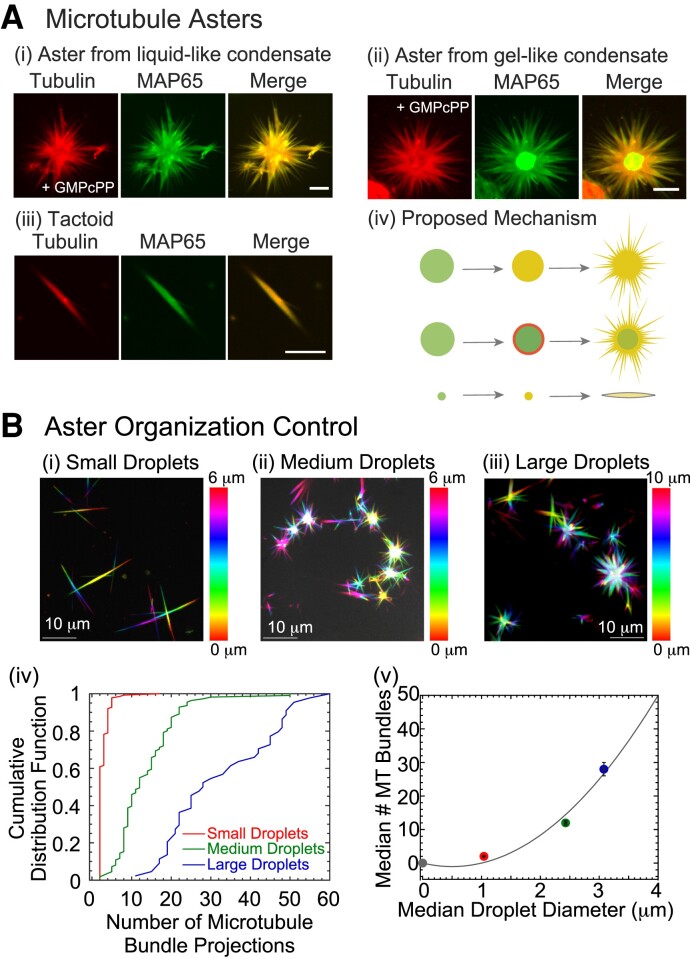
MAP65 droplets organize microtubule bundles into asters and tactoids. A) Microtubules nucleate and grow bundles from MAP65 condensates. (i) Maximum projection of confocal z-stack for microtubule aster formed from liquid-like condensate showing tubulin (left), GFP-MAP65 (middle) and merge (right). (ii) Maximum projection of confocal z-stack for microtubule aster formed from gel-like condensate showing tubulin (left), GFP-MAP65 (middle), and merge (right). (iii) Maximum projection of confocal z-stack for microtubule tactoid formed from small condensate showing tubulin (left), GFP-MAP65 (middle) and merge (right). Scale bars are 5 μm for all images. (iv) Cartoon schematic of aster and tactoid formation. B) Confocal z-stack images of microtubule organizations in the presence of (i) small droplets with median diameter of 1.04±0.01μm for 1μM, (ii) medium droplets with median diameter of 2.43±0.02μm for 5μM, and (iii) large droplets with median diameter of 3.08±0.02μm for 10μM. Color coding for all images based on z-height given by color bars; scale bar is 10 μm. (iv) Cumulative probability distribution function of the number of microtubule bundle projections for small (red line, left-most curve), medium (green line, center curve), and large (blue line, right-most curve) droplets. (v) The median number of microtubule bundle projections plotted against the median diameter of the MAP65 condensates to show a quadratic relationship, fit with a parabola (fit parameters in Supplementary material). Error bars represented the standard error of the mean for the droplet diameter (horizontal) and the number of projections (vertical).

Next, we examine if the tubulin co-localization can nucleate and grow microtubules from the condensate. We add GMPcPP and the same tubulin concentration to preformed droplets at [MAP65]=28μM. We specifically use the slowly hydrolyzable analog of GTP to nucleate, stabilize, and grow microtubules in order to control the length of the filaments, as previously shown (6, 8, 9, 45). For both liquid-like and gelated droplets, the tubulin accumulates in the droplet and can nucleate and grow microtubules that form projected microtubule bundles (Fig. 4A). These projections are finite sized and tapered. The microtubule bundles co-localize exactly with MAP65 (Fig. 4A). For gel-like MAP65 condensates, there is a core of higher concentration MAP65 where fewer microtubules are formed presumably due to the lower tubulin concentration (Fig. 4Aii). For both types of droplets, the MAP65 is found to distribute along the microtubules of the aster.

In addition to asters with many projecting microtubule bundles, we also observed microtubule tactoids in the same samples (Fig. 4Aiii). Tactoids are finite sized, tapered bundles of microtubules which we have previously observed when microtubules were nucleated in the presence of MAP65 (9, 29). Tactoids appear to be nucleated and grown from smaller MAP65 droplets, which likely only support bidirectional microtubule growth.

The data imply that the the microtubule organization, specifically, the number of projected microtubule bundles, might be controlled by the droplet size. To test this, we use low, medium, and high MAP65 concentrations of 1, 5, and 10 μM, to create droplets that have median (same as mean) diameters of 1, 2.5, and 3 μm, respectively (Fig. 1, D–F). When these droplets are added to 3.75 μM tubulin with GMPcPP, they are able to polymerize tactoids on small droplets and asters on large droplets, as expected (Fig. 4Bi–iii). The median number of projections quantified for small droplet samples is 2, which increases to 12 and 28 projections for medium and large droplets (Fig. 4Biv–v). We plot the median number of microtubule projections as a function of the median droplet diameter and find a quadratic relationship (Fig. 4Bv, Supplementary material). The quadratic relationship is reasonable, since we would expect the number of arms to grow with the surface area of the droplet, which also scales as radius squared. These data suggest that the MAP65 droplet size can directly control the spatial organization of microtubule asters, specifically controlling the number of microtubule bundle projections from the droplet.

### Microtubules can recover fluidity of gelated MAP65 condensates

**Fig. 5.**
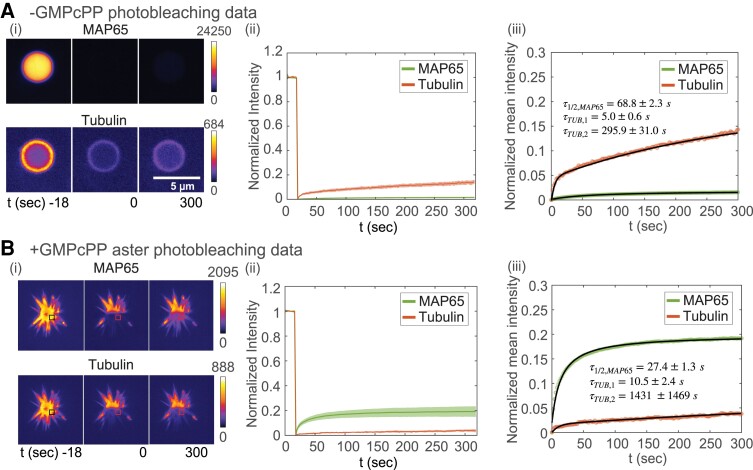
Photobleaching experiments on gel-like MAP65 condensates in presence of tubulin without and with nucleotide to form microtubules. A) FRAP on condensate. (i) Time series images of whole droplet FRAP for time points at t=−18, 0, and 300 s after photobleaching for GFP-MAP65 (top) and rhodamine-tubulin (bottom). Intensity portrayed using fire look up table, as denoted by color bar and ranges given. Scale bar is 5 μM. (ii) Normalized FRAP recovery curves for GFP-MAP65 (green, lower curve) and rhodamine-tubulin (red, upper curve). The shaded region denotes the standard deviation around the mean (N=5). (iii) Normalized mean intensity curves fit with Eq. [1] in Supplementary material for GFP-MAP65 (green data, black line, lower curve) or biphasic exponential recovery for tubulin (red data, black line, upper curve). B) FRAP on asters. (i) Time series images of partial aster FRAP for time points at t=−18, 0, and 300 s after photobleaching for GFP-MAP65 (top) and rhodamine-tubulin (bottom). Intensity portrayed using fire look up table, as denoted by color bar and ranges given. Boxes represent area where analysis was performed. Scale bar is 5 μM. (ii) Normalized FRAP recovery curves for GFP-MAP65 (green, upper curve) and rhodamine-tubulin (red, lower curve). The shaded region denotes the standard deviation around the mean (N=6). (iii) Normalized mean intensity curves fit with Eq. [1] in Supplementary material for GFP-MAP65 (green data, black line, upper curve), or biphasic exponential recovery for tubulin (red data, black line, lower curve).

To examine the mobility of each species in the condensates with tubulin, we perform two-color FRAP on gelated MAP65 droplets that co-localized tubulin dimers or nucleated asters (Fig. 5Ai). In the absence of nucleotide, MAP65 recovery is minimal, with a smaller mobile fraction (2%) and similar recovery time compared to what was measured for gel-like MAP65 condensates without tubulin (compare Fig. 5Aii to Fig. 2). The tubulin FRAP shows an interesting biphasic recovery. We fit the normalized mean intensity with a double exponential equation, $I_{TUB}(t)=A(1-e^{-t/\tau_{1}})+B(1-e^{-t/\tau_{2}})$. The fast timescale is on the order of 5 s, and the slower recovery is 300 s (Fig. 5Aiii). We also test if tubulin is comparatively less mobile at the center of the droplet by quantifying the recovery rates in the interior compared to the edge of the droplet. We find the same biphasic recovery for the interior and the boundary of the condensate, with longer timescales for interior tubulin which mixes at a slower rate compared to the tubulin at the boundary of the MAP65 droplet (Supplementary Material, Fig. S8).

Next, we perform FRAP on microtubule asters formed around gel-like MAP65 condensates (Fig. 5B). We find that the mobile fraction of MAP65 is an order of magnitude higher and the recovery timescale is two times faster in the presence of the microtubules (Fig. 5Biii). The tubulin, which is now polymerized into microtubules, is less mobile in asters, and the recovery is still biphasic. The mobile fraction drops by a factor of three, the faster timescale increases by a factor of two, and the longer timescale increases by a factor of five (Fig. 5Biii). This result is surprising, since the aster is formed around an aged MAP65 condensate that has low MAP65 mobility when unpolymerized tubulin is present, indicating that the formation of the microtubules can release MAP65 from the gel-like core and alter the phase of the condensed MAP65. This phenomena is not a product of the tubulin, but requires microtubules to nucleate and grow.

## Discussion

In this study, we show that the microtubule-associated proteins, MAP65 and PRC1, which are known to crosslink and nucleate microtubules (24, 26, 46, 47), are both able to form condensates at physiological conditions of low salt, moderate temperature without crowding agents in vitro (Fig. 1, Supplementary material, Figs. S2–S4), which is different from other MAPs, such as tau (16, 48). The MAP65 condensates are liquid-like and change their material properties to become gel-like over the course of several hours. The gel-like state is never fully solid since there is still a mobile fraction (Fig. 2). This implies that the core of the mature condensate is likely a viscoelastic network of MAP65 molecules, as opposed to a crystaline solid or a rigid aggregate.

Regardless of the material properties of the MAP65 condensates, condensed droplets are able to co-localize high concentrations of tubulin to cause growth of microtubules into asters (Fig. 4A). The size of the droplets can control the organization of the microtubules, specifically, the number of bundles scales with the surface area of the droplet. Excitingly, the presence of the microtubules growing from the condensate can reverse the gelation, refluidizing the crosslinker molecules (Fig. 5).

The ability of the MAP65 condensates to localize higher concentrations of tubulin, enough to cause the localized nucleation and growth of microtubule bundles and asters, could be a biologically relevant method for controlling the spatial organization in cells. In mammalian cells, PRC1 could help to nucleate and grow the overlapping interpolar array of microtubules that are needed to push apart the choromosomes during mitosis. Indeed, a recent paper has shown that PRC1 along with Kif4A can compact microtubules (47). The bundles created in that study look similar to the tactoids we observe created from small droplets, and it is possible that the initial organizations at early times are formed from small condensates that were not observed due to initially mixing all the proteins together.

MAP65 condensates of liquid or gel-like states could serve as small microtubule-organizing centers in plant cells to create the bundles and arrays needed for cell division and cellulose deposition. Indeed, MAP65 family proteins are important for creating the phragmoplast in plant cells during mitosis (49, 50) and directing the cortical microtubule array in plants (24, 51). Our work implies that these organizations could initiate from small condensed droplets of MAP65 that can nucleate antiparallel bundles, as long as there was at least 50 nM MAP65. In cells, crowding and co-localization with other molecules can increase local concentrations further, nucleating and growing microtubules, controlled in space and time. Based on the concentrations expected in cells, these organizations would most likely be bundles, and not asters, since we show that small droplets form bundles with two microtubule projections (Fig. 4). Further, the initial condensates of MAP65 or PRC1 would likely form and immediately initiate the nucleation and growth of microtubules due to the high cellular concentration of tubulin, which is close to 5 μM (28), making these small condensates difficult to observe and quantify.

The use of cytoskeletal-associated proteins to form condensates that can direct the organization of microtubules and actin is likely a general organizational principle in cells. Indeed, cytoskeletal fiber formation from condensates has recently been reported for numerous cellular and in vitro studies for microtubules (16, 18, 20) and actin (52–55). This may suggest that cells use this universal strategy to control cytoskeletal filament organization (56), but it is especially important for cell-types or regions of the cell that do not have centrioles to nucleate and grow microtubules in the traditional manner. Further, demonstrating that the gelation of these condensates is not a barrier to nucleation and growth makes the condensate-based cytoskeletal organzation mechanism more useful and applicable.

As with many biological systems, there appears to be a feedback loop that the growth of microtubules from gelated MAP65 condensates can refluidize the condensate and release the MAP65 back into the solution. In the gelated condensates, MAP65 molecules likely bind to each other to form a polymer network inside the less mobile core. We postulate that when microtubules form, they reduce the MAP65–MAP65 interactions in favor of MAP65–microtubule interactions. Further, although microtubules appear excluded from the condensate core, the microtubules at the edge are likely able to draw out and exchange MAP65 at the condensate edge, increasing the mobile fraction and decreasing recovery time (Fig. 5). Thus, the microtubule organization can regulate the condensate. Overall, our work demonstrates that microtubule-associated proteins and crosslinkers can regulate microtubule organization and, in turn, be regulated by the microtubules.

## Materials and methods

**Protein reagents.** MAP65 constructs were gifts from Ram Dixit (Washington University, St. Louis). MAP65 and GFP-MAP65 are expressed in *Escherichia coli* BL21(DE3) cells and purified using 6× His-tag affinity purification (9, 57).

Unlabeled and rhodamine-labeled lyophilized tubulin from porcine brain was purchased from Cytoskeleton. Lyophilized tubulin powder was resuspended in PEM-80 buffer (80 mM PIPES, pH 6.8, 1 mM MgCl${}_{2}$, 1 mM EGTA), aliquoted, drop-frozen, and stored at −80${}^{\circ }C$ for later use.

**Phase separation and aster assays.** Condensates were formed by adding the specified amount of protein in PEM-80 buffer at 22${}^{\circ }C$ and incubating at the specified temperature prior to imaging. No crowding agents were used in the buffer. Imaging chambers were made using silanized cover slips with Pluronic F127 polymer coatings, as previously described (9). Chambers could be made from permanent double-sided tape or by using epoxy to attach a reaction cylinder to the cover glass. For asters, droplets were added to tubulin solutions in reaction chambers allowing droplets to fall to the cover slip.

**Confocal imaging.** Condensates and asters were imaged using spinning disc microscopy (Yokogawa CSU-W1) on an inverted Nikon Ti-E microscope with Perfect Focus and 100x oil immersion objective (1.49 NA) imaged onto a Andor Zyla CMOS camera. Images and image sequences were captured and saved as .nd2 files which are stacks of tifs with metadata.

**Image analysis.** Images and image sequences were opened using the Bioformats Importer plugin in ImageJ/FIJI. Further, the images were preprocessed, analyzed, and plotted using MATLAB. Complete details of the analysis for photobleaching and mechanical measurements are provided in the Supplementary material.

## Supplementary Material

pgad231_Supplementary_Data

## Data Availability

Original data created for the study are available in a persistent repository upon publication at this DOI: https://doi.org/10.5281/zenodo.8128492
